# Systemic sodium hydrosulfide (NaHS) administration mitigates reperfusion injury in an experimental model of ischemic priapism

**DOI:** 10.1007/s00345-026-06349-6

**Published:** 2026-03-16

**Authors:** Nilay Kaya, Emine Nur Ozbek, Nevin Ersoy, Gunay Yetik-Anacak, Serap Cilaker Micili, Ozan Bozkurt, Nergiz Durmus

**Affiliations:** 1 Health Sciences Institute, Dokuz Eylul University, Izmir, Türkiye; 2 Faculty of Pharmacy, Department of Pharmacology, Ege University, Izmir, Türkiye; 3 Faculty of Medicine, Department of Histology and Embryology, Izmir Tınaztepe University, Izmir, Türkiye; 4 Faculty of Pharmacy, Department of Pharmacology, Acibadem Mehmet Ali Aydinlar University, Istanbul, Türkiye; 5 Faculty of Medicine, Department of Histology & Embryology, Dokuz Eylul University, Izmir, Türkiye; 6 Faculty of Medicine, Department of Urology, Dokuz Eylul University, Izmir, Türkiye; 7 Faculty of Medicine, Department of Medical Pharmacology, Dokuz Eylul University, Izmir, Türkiye

**Keywords:** Hydrogen sulfide, Hypoxia-Inducible Factor-1 alpha (HIF-1α), Ischemia-reperfusion injury, Priapism

## Abstract

**Purpose:**

Ischemic priapism (IP) is characterised by prolonged, painful erections that require urgent intervention to prevent corporal fibrosis and erectile dysfunction. Hydrogen sulfide (H₂S), a gaseous mediator, has been shown to exert protective effects against ischemia-reperfusion (IR) injury by reducing oxidative stress, enhancing mitochondrial function, and inhibiting apoptosis. This study aimed to investigate the role of H₂S during IP and reperfusion and its potential protective effects against penile tissue injury.

**Methods:**

Twenty-eight male Wistar rats were divided into four groups: control, IP, IP-R, and IP-[NaHS]-R. The control group underwent penectomy only. In the IP group, penectomy was performed after 4 h of priapism, whereas in the IP-R group, it was performed after 4 h of priapism followed by 1 h of reperfusion. In the IP-[NaHS]-R group, rats received NaHS (75 µmol/kg, intraperitoneally) 10 min before reperfusion. Endogenous H₂S levels were determined by the methylene blue assay, and hypoxia-inducible factor-1α (HIF-1α) expression was evaluated by immunohistochemistry.

**Results:**

Histopathological analyses were performed to assess tissue alterations. The IP and IP-R groups showed edema, inflammation, desquamation, vasocongestion, and increased collagen deposition, and exhibited reduced H₂S levels (*p* < 0.05) and elevated HIF-1α (*p* < 0.0001) compared with control group. NaHS administration increased H₂S levels, reduced HIF-1α expression, and alleviated histopathologic changes.

**Conclusion:**

Decreased H₂S levels during ischemia and reperfusion were associated with penile tissue injury in IP. NaHS mitigated morphological damage during the initial phase of reperfusion, indicating the potential involvement of H₂S signaling in reperfusion-related corporal tissue injury. However, endpoints related to the resolution of priapism, such as detumescence time and intracavernosal pressure (ICP), have not been evaluated. These assessments are necessary to determine the translational safety and therapeutic applicability of these treatments in cases of IP.

## Introduction

Priapism is a persistent and painful erection lasting more than four hours without sexual stimulation, significantly impairing quality of life. Ischemic priapism (IP), the most common type, accounts for nearly 95% of cases [1]. IP requires urgent intervention, typically beginning with aspiration and intracavernous injection of sympathomimetic agents; surgery may be necessary if these fail [2]. Prolonged erection leads to reduced oxygenation, hypercarbia, and acidosis in the erectile tissue [3]. Hypoxia activates endothelial cells, leading to increased neutrophil adhesion, decreased mitochondrial respiratory activity, and elevated intracellular calcium levels. During ischemia, antioxidant defences decline, impairing mitochondrial metabolism and promoting the formation of reactive oxygen species (ROS). The nitric oxide (NO) pathway contributes to oxidative stress through the generation of peroxynitrite and hydroxyl radicals [4]. Decreased Rho/Rho-kinase activity is also implicated in IP pathophysiology [5]. Although reperfusion restores oxygen supply, it paradoxically increases ROS, aggravating tissue damage [6]. Elevated adenosine levels in hypoxic penile tissue further exacerbate IP by suppressing PDE5 gene expression via HIF-1α signalling [7].

Hydrogen sulfide (H₂S) is another gasotransmitter involved in penile smooth muscle relaxation that occurs following NO [8]. Beyond its role in erectile physiology, H₂S has been shown to modulate ischemia-reperfusion injury. Studies reveal that H₂S levels decrease during ischemia–reperfusion in myocardial tissue and plasma, while exogenous H₂S administration reduces oxidative stress and apoptosis [9]. Its cytoprotective effects have been demonstrated in multiple organs, including the heart, brain, liver, kidneys, lungs, testes, and the corpus cavernosum [10, 11].

This study aimed to evaluate the role of H₂S in a rat model of IP , with a specific focus on the reperfusion period, to investigate its potential involvement in attenuating reperfusion-induced penile tissue injury.

## Materials and methods

### Animals

All procedures were approved by the Institutional Animal Care and Use Committee (Approval No: 20–2021). Twenty-eight adult male Wistar rats (250–300 g) were used. Animals were housed in identical cages at 23 °C with a 12-h light–dark cycle and had ad libitum access to standard chow and water. Experiments were performed under urethane anaesthesia (1200 mg/kg, administered intraperitoneally). At the end of the study, all rats were euthanised by cervical dislocation.

### Experimental study design

#### Induction of ischemic priapism

**Fig. 1:**
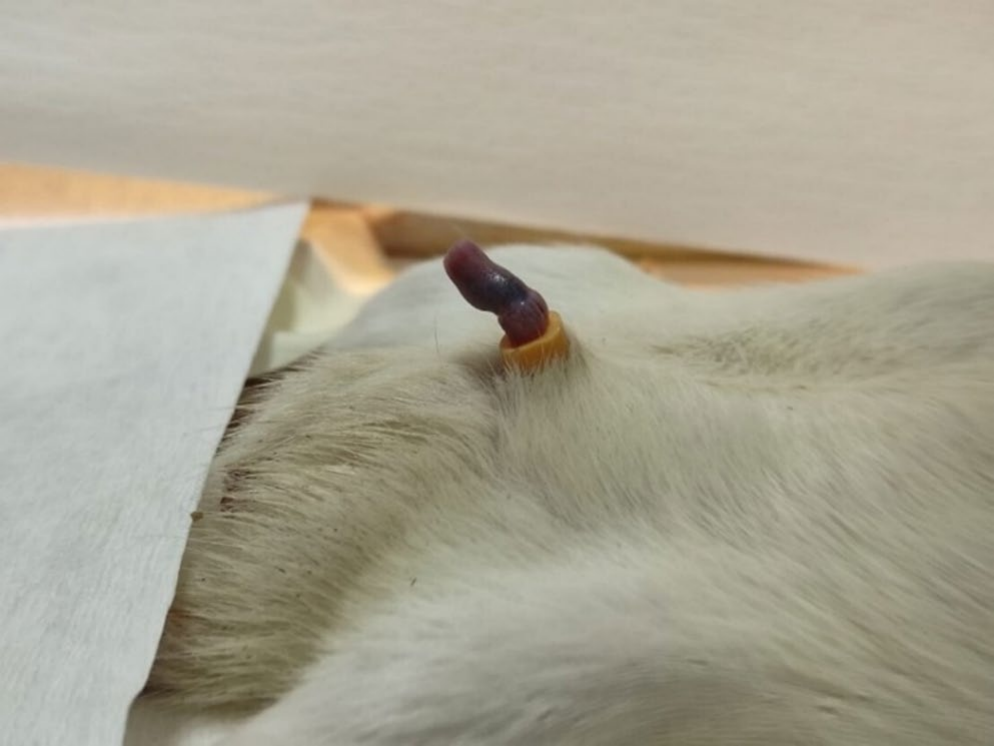
Induction of erection and priapism model

Ischemic priapism (IP) was induced using a negative-pressure vacuum method. After prepuce retraction, a 50-mL syringe with a cone-shaped tip was positioned at the penile base, and negative pressure was applied by slowly pulling back the plunger until an erection occurred (10 ± 1 s) (Fig. 1). A 2-mm–long segment cut from a 16G Foley catheter was placed around the proximal penis to occlude venous return for 4 h [12]. This duration was selected based on previous reports indicating irreversible smooth muscle injury and acidosis after 4–6 h of ischemia [13].

#### Experimental groups

**Fig. 2:**
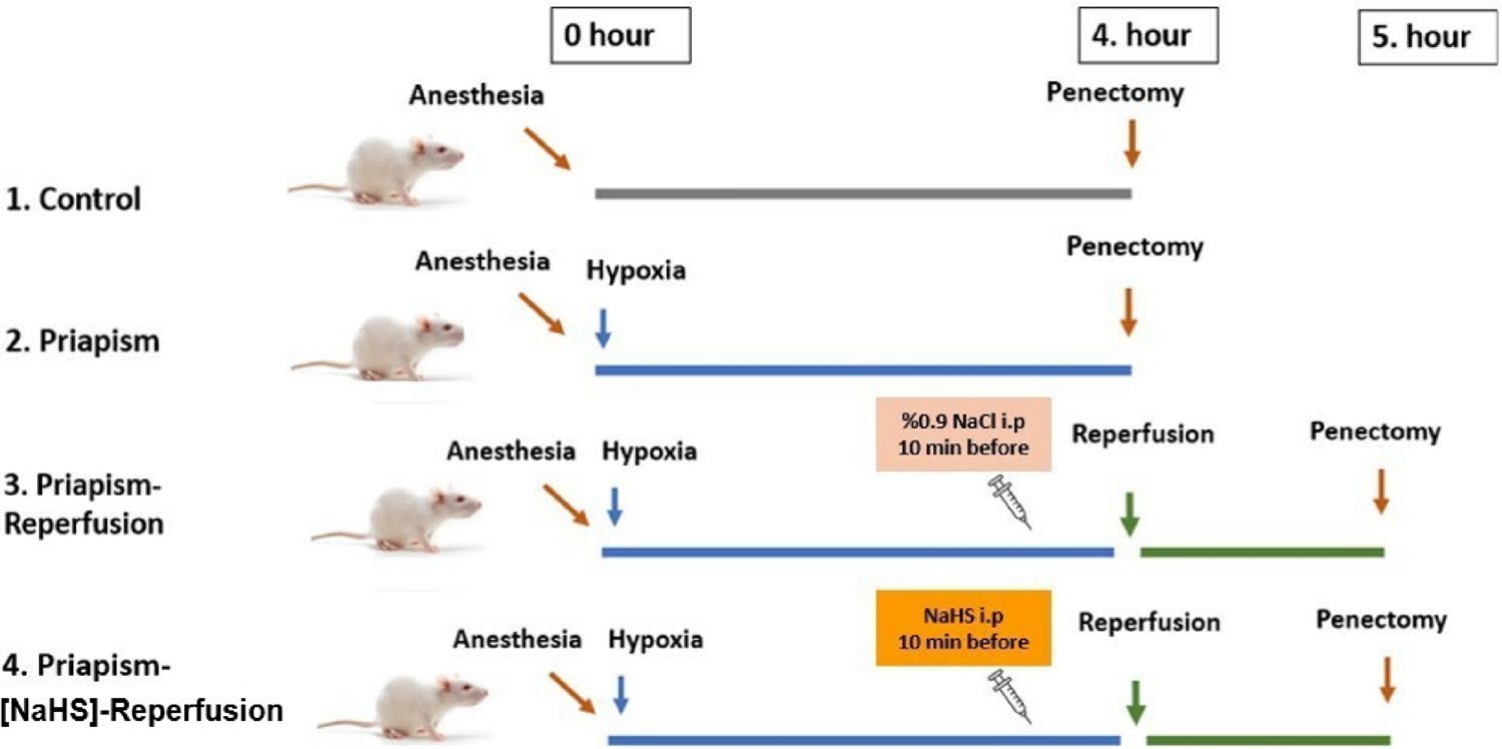
Study protocol

Animals were randomly assigned to the experimental groups at the time of group allocation. Four experimental groups were formed, with seven animals in each group. Control: anaesthesia only (no intervention). IP: 4 h of induced priapism. IP-R: 4 h of ischemia followed by 1 h of reperfusion. IP-[NaHS]-R: 4 h of ischemia followed by 1 h of reperfusion, with NaHS (75 µmol/kg, intraperitoneally) [14] administered 10 min before reperfusion (Fig. 2). The required amount of NaHS was individually calculated according to each animal’s body weight and dissolved in physiological saline to a final injection volume of 5 mL/kg prior to intraperitoneal administration [15].

At the end of the experimental protocol, penile tissues from the experimental groups were excised and divided into proximal and distal segments. Samples from both regions were collected for H₂S measurement, histopathological evaluation, and HIF-1 analysis. Samples for H₂S assays were stored at − 80 °C, while tissues for histopathological and immunohistochemical analyses were fixed in 10% neutral-buffered formalin.

### Tissue homogenization and measurement of endogenous H₂S production

Penile cavernous tissues (40–50 mg) from 7 animals per groups homogenised in 0.1 mM potassium phosphate buffer (PPB, pH 7.4) containing protease and phosphatase inhibitors using a cryogenic homogeniser under liquid nitrogen. Protein concentrations in the homogenates were determined using the bicinchoninic acid (BCA) method.

Endogenous H₂S production was quantified using the methylene blue assay (MBA) as previously described [16, 17]. Tissue homogenates (50 µg protein) were incubated in sealed tubes at 37 °C for 30 min with L-cysteine (10 mM) and pyridoxal phosphate (2 mM) to stimulate H₂S synthesis. Basal samples were incubated without L-cysteine. Standard curves were prepared using NaHS dilutions (250–3.9 µM).

After incubation, 10% trichloroacetic acid was added to stop the reaction, followed by 1% zinc acetate to trap H₂S as zinc sulfide. Subsequently, N,N-dimethyl-p-phenylenediamine sulfate (20 mM) and FeCl₃ (30 mM) were added to generate methylene blue. After 15 min in the dark, 200 µL aliquots were transferred to a microplate, and absorbance was measured at 650 nm using a spectrophotometer. Each group was measured in triplicate. H₂S production was calculated from the standard curve and expressed as nmol H₂S produced per minute per mg of protein (nmol·min⁻¹·mg⁻¹ protein).

### Histopathological examination

Penile tissues were fixed in 10% formalin solution. After routine tissue processing, the sample was embedded in parafine and 5 μm sections were taken (RM 2255, Leica.). Slides were stained with hematoxylin–eosin (H&E) to evaluate general morphology and with Masson’s trichrome to assess collagen deposition. Tissues were examined by light microscopy (Euromax, Iscope, Holland) and evaluated by two histologist who was blinded to the experimental design.

Histological evaluation was performed under a light microscope and at 40× magnification. The presence of vasocongestion, inflammation, desquamation, and edema was scored between 0 and 3 points as follows: 0: normal, 1: mild, 2: moderate, 3: severe [18]. Vasocongestion, inflammation, and edema were assessed within the corpus cavernosum stroma, including the cavernous sinusoids and trabecular structures, as well as in the tunica albuginea and corpus spongiosum, whereas desquamation was evaluated exclusively in the urethral epithelium. Vasocongestion was defined as dilation of cavernous sinusoids accompanied by intravascular erythrocyte accumulation and blood pooling. Inflammation was identified based on the presence and extent of inflammatory cell infiltration within interstitial and perivascular areas. Desquamation was assessed as detachment or loss of epithelial cells, and edema was evaluated according to the degree of interstitial space expansion and tissue loosening.

Masson’s trichrome (MT) staining were graded on a scale of (+) to (++++) according to the percentage of collagen in the penile corpus cavernosum in each group: as follows: +, 30% or less collagen; ++, 30–50% collagen; +++, 50–70% collagen; and ++++, more than 70% collagen [19].

For histological evaluation, (H&E) and MT sections were analyzed by selecting five randomly chosen, non-overlapping fields per animal, and scoring was performed for each field. The mean score of these fields was calculated to obtain a representative value for each subject.

### Immunohistochemical detection of HIF-1α

Paraffin sections were deparaffinized, rehydrated, and subjected to heat-induced epitope retrieval in 10% citrate buffer (pH 6.0) for 5 min. Endogenous peroxidase activity was blocked with 0.3% hydrogen peroxide for 10 min. Slides were incubated overnight at 4 °C with anti-HIF-1α primary antibody (BS-0737R, Bioss, USA) at a dilution of 1:100. After washing, sections were treated with a ready-to-use streptavidin–biotin secondary antibody complex (Invitrogen) for 30 min and visualised using diaminobenzidine (DAB) as the chromogen. Counterstaining was performed with Mayer’s hematoxylin, followed by dehydration and mounting. HIF-1α immunoreactivity was evaluated by light microscopy. Immunohistochemical evaluation was undertaken using the semi-quantitative H-score method. The intensity of positive cell staining (i value) was categorized as 0 (no staining), 1 (weak but detectable staining), 2 (moderate staining), and 3 (intense staining). Five randomly selected fields were scanned microscopically at 40x magnification, and the average of these scores was used for statistical analysis. The sum of their percentages was calculated according to the following formula; H-Score = ∑Pi(i + 1). According to the formula, i is the intensity of staining at values ​​of 1, 2, or 3 (weak, moderate, or strong, respectively), and Pi is the percentage of stained cells for each intensity, ranging from 0% to 100% [20].

HIF-1α immunoreactivity was observed in both the urethral epithelium and corpus cavernosum in representative sections, semiquantitative scoring revealed comparable staining intensity and distribution in these regions across all groups; therefore, regional separation was not applied in the final evaluation [21].

### Statistical analysis

Data were analysed using GraphPad Prism 8 (GraphPad Software, San Diego, USA). The Shapiro-Wilk test was used to evaluate whether continuous variables were normally distributed. Parameters with a normal distribution were compared using one-way ANOVA followed by Bonferroni’s post hoc test, while those not normally distributed were analysed using the Kruskal–Wallis test, pairwise comparisons performed using the Mann–Whitney U test. Values were expressed as mean ± SEM, and *p* < 0.05 was considered statistically significant. Group sample size (n) refers to the number of animals per condition.

## Results

### Histological evaluations

**Fig. 3:**
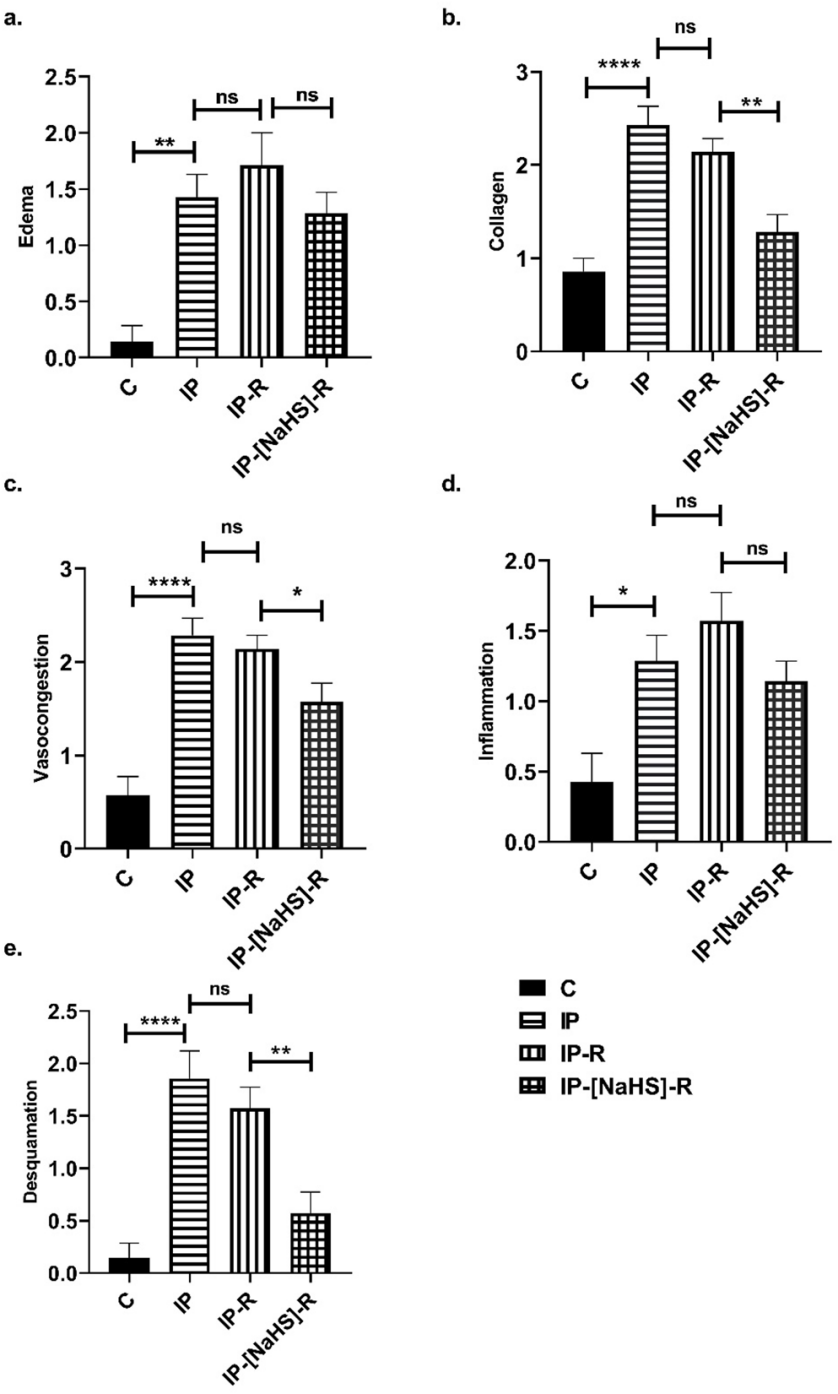
Comparison of histopathological scores of penile tissue damage between groups. The evaluated parameters are **a** Edema, **b** Collagen, **c** Vasocongestion, **d** Inflammation, **e** Desquamation. (Control (C), Ischemic Priapism (IP), Priapism-Reperfusion (IP-R), and Priapism-NaHS-Reperfusion (IP-[NaHS]-R). Statistical significance is indicated as follows: *p < 0.05, **p < 0.01, ****p < 0.0001, and “ns” means no significant difference, *n* = 7

**Fig. 4:**
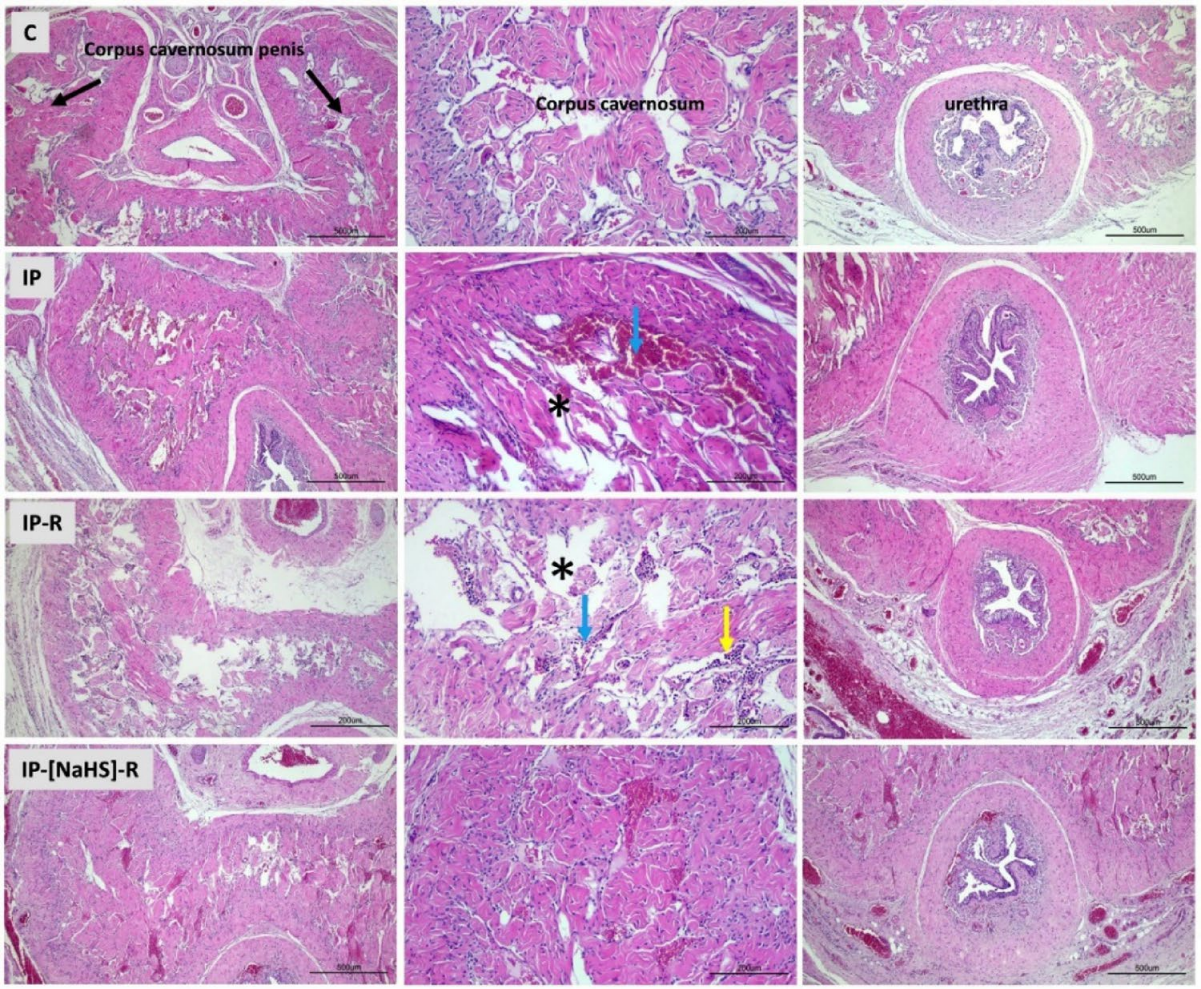
Light microscopic images of penile tissue from the experimental groups. In hematoxylin and eosin–stained sections, the structures of the penile corpus cavernosum (CC), urethra, and corpus spongiosum appeared normal in the control group. In the IP group, widespread vascular congestion (*), hemorrhage (blue arrow), and occasional degeneration of the urethral epithelium were observed in the central and peripheral regions of the CC. The IP-R group showed vascular congestion (*) and inflammatory cell infiltration (yellow arrow) in the CC. The IP-[NaHS]-R group exhibited histological features similar to those of the control group. (Hematoxylin and eosin staining; scale bar: 200–500 μm; magnification: ×10–×40.)

**Fig. 5:**
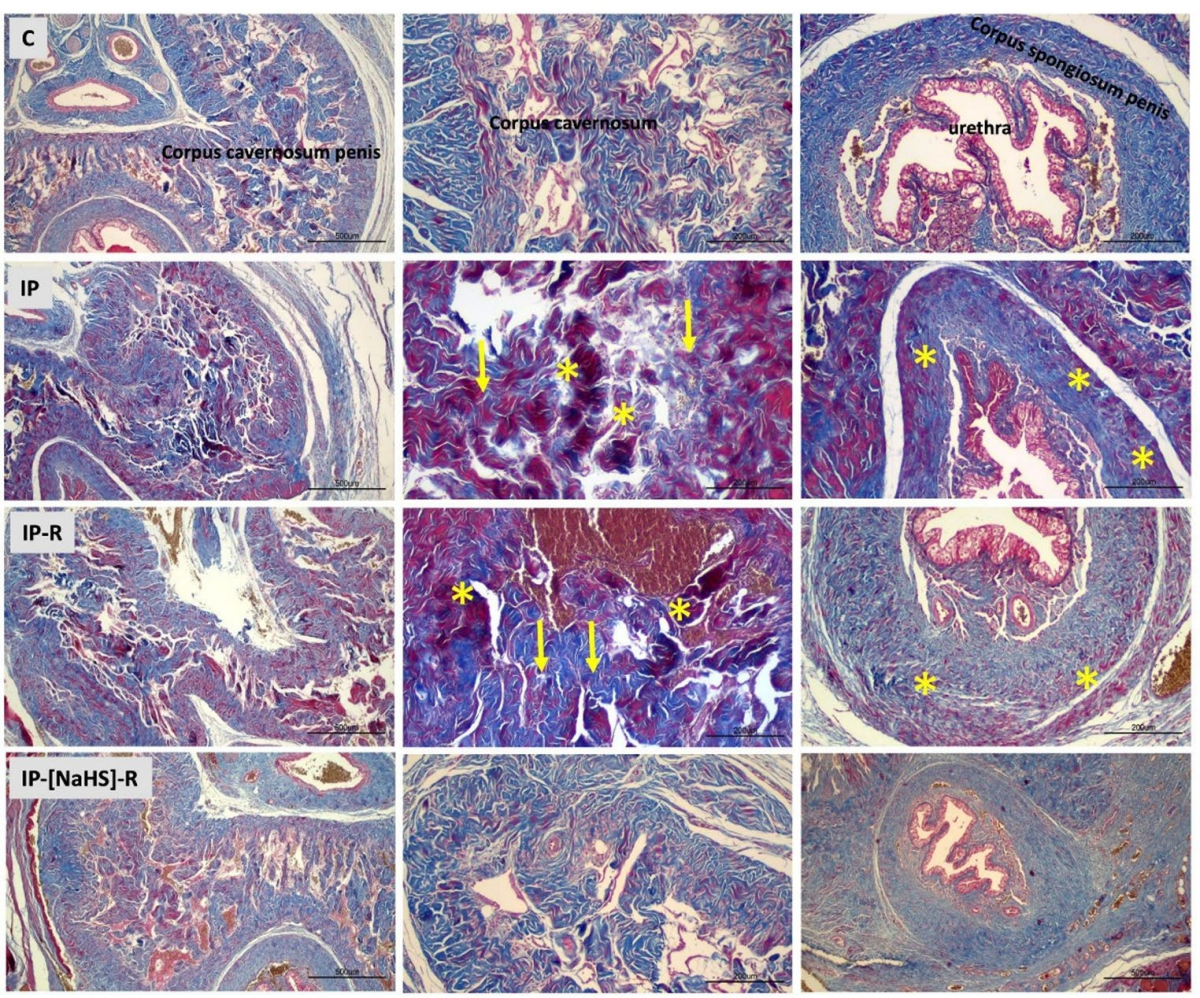
Light microscopic images of Masson’s trichrome–stained penile tissue from the experimental groups. In the control group, connective tissue and smooth muscle structures within the cavernous tissue of the penis appeared normal. In the IP group, increased smooth muscle content (*) and collagen deposition (yellow arrow) were observed in the cavernous tissue. The IP-[NaHS]-R group exhibited histological features similar to those of the control group. (Masson’s trichrome staining; scale bar: 200–500 μm; magnification: ×10–×40.)

This study evaluated histological changes in penile tissues caused by priapism and examined the influence of reperfusion on these changes. In addition, the effects of NaHS administration during the reperfusion period were investigated, with a focus on vascular congestion, desquamation, collagen alterations, inflammation, and edema. An increase in vascular congestion was observed in both the central and peripheral cavernous tissues in the IP group compared to the control group (*p* < 0.0001). Vasocongestion observed in the IP-R group did not differ from that in the IP group (*p* > 0.05). The NaHS-treated IP-R group exhibited a statistically significant decrease in vascular congestion compared with the IP-R group (*p* < 0.05) (Figs. 3 and 4). An increase in desquamation of the urethral epithelium was observed in association with priapism (*p* < 0.0001). Desquamation in the reperfusion group did not significantly differ from that in the priapism group (*p* = 0.9086). However, in the IP-NaHS-R group, urethral epithelial desquamation was significantly decreased compared with the IP-R group (*p* = 0.0044; Figs. 3 and 4). Regarding inflammation, the IP group showed a significant increase compared with the control group (*p* = 0.0191). Inflammation tended to be higher in the IP-R group than in the IP group; however, the difference was not statistically significant (*p* = 0.9513). NaHS administration before reperfusion did not reduce inflammation during reperfusion (*p* = 0.1089) (Figs. 3 and 4). Priapism-induced edema differed significantly from that in the control group (*p* = 0.0017) and persisted during the reperfusion period, but this persistence was not statistically significant (*p* > 0.05). NaHS treatment did not result in a statistically significant reduction in edema in the cavernous tissue compared with the IP-R group (*p* = 0.2315; Figs. 3 and 4). A significant increase in collagen levels was observed in the cavernous tissue in response to priapism (*p* < 0.0001); however, the elevation did not remain significant during the reperfusion period (*p* = 0.7094). Collagen levels were significantly decreased in the IP-[NaHS]-R group compared with the IP-R group (*p* = 0.0032; Figs. 3 and 5).

### Hypoxia-inducible factor-1 alpha levels

**Fig. 6:**
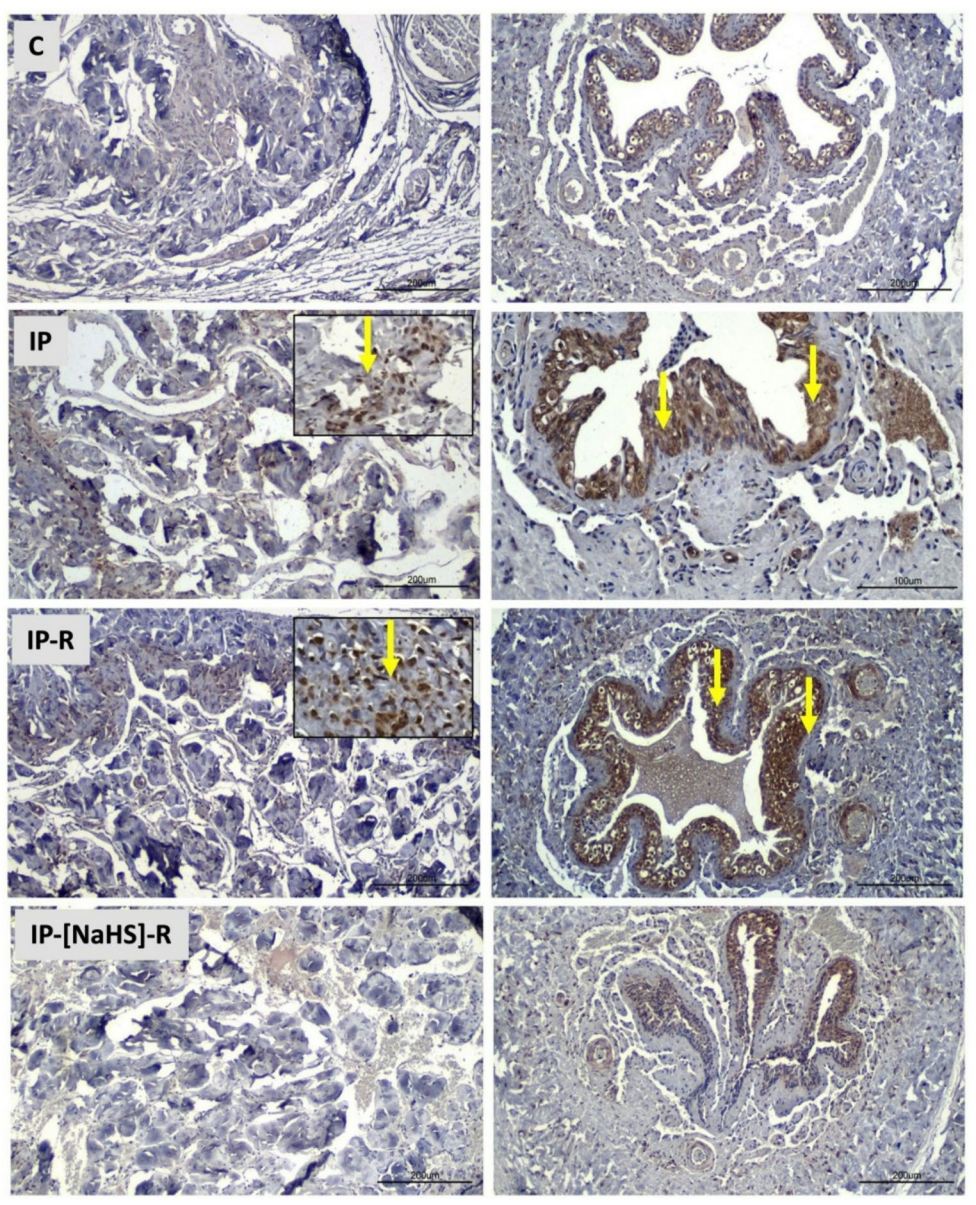
Light microscopic images of HIF-1α immunohistochemical staining in penile tissue from the experimental groups. Yellow arrows indicate immunopositive cells. Increased HIF-1α immunoreactivity was detected in both the cavernous tissue and urethral epithelium in the IP and IP-R groups. In contrast, the IP-[NaHS]-R group showed a reduced number of positive cells, comparable to the control group. (Scale bar: 200 μm; magnification: ×40.)

**Fig. 7:**
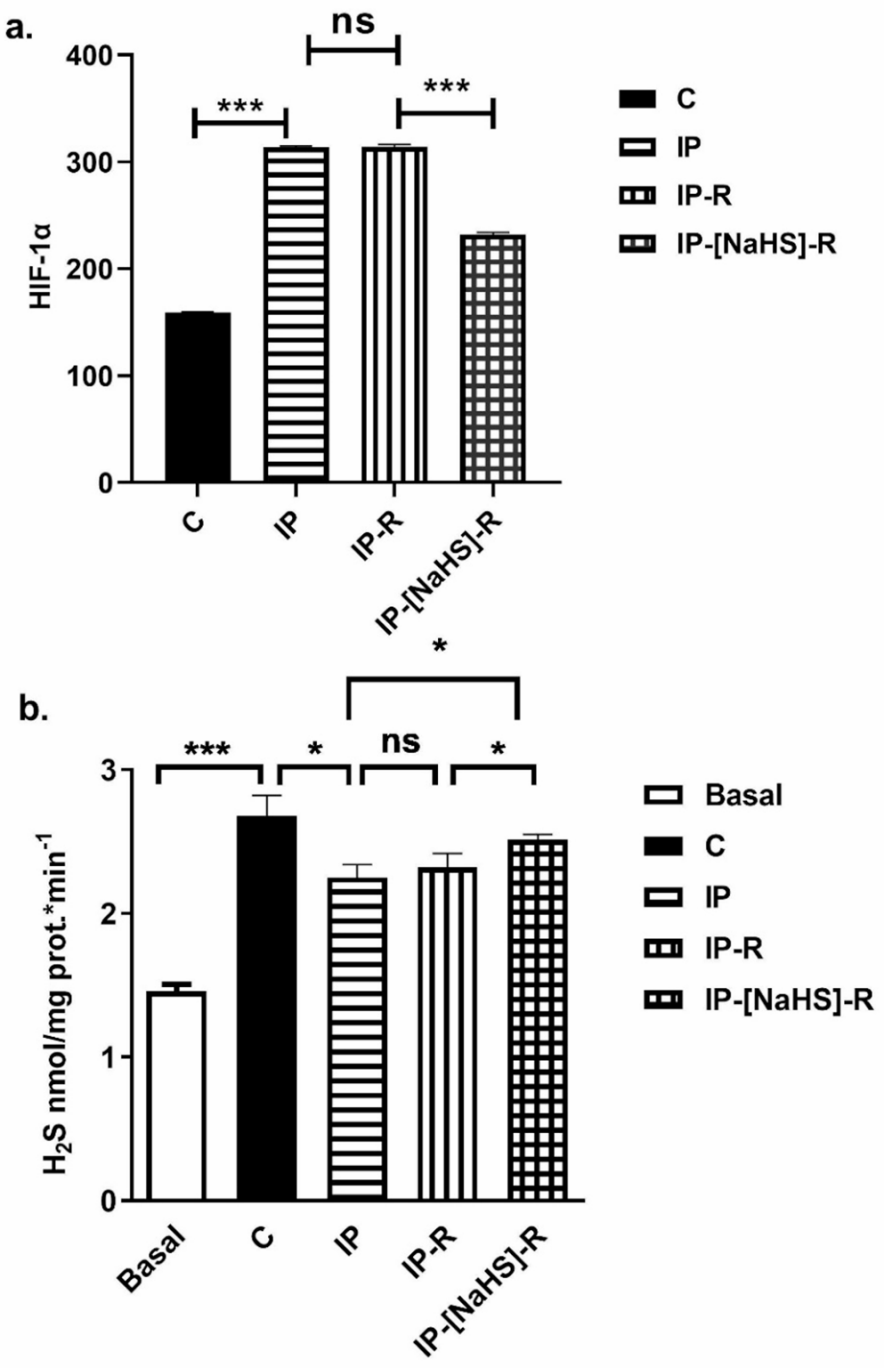
Effects of NaHS treatment on **a** HIF-1α levels and **b** endogenous H₂S levels in penile tissue homogenates from the control (C), ischemic priapism (IP), ischemic priapism–reperfusion (IP-R), and ischemic priapism–NaHS–reperfusion (IP-[NaHS]-R) groups. (* *p* < 0.05, *** *p* < 0.001, one-way ANOVA followed by Bonferroni’s *post hoc* test; *n* = 7)

A statistically significant increase in HIF-1α levels was detected in the IP group compared with the control group (*p* < 0.0001), whereas HIF-1α levels in the IP-R group did not differ significantly from those in the IP group (*p* > 0.99). NaHS treatment decreased HIF-1α levels during reperfusion. (*p* < 0.0001) **(**Figs. 6 and 7a**).**

### Hydrogen sulfide levels

As expected, L-cysteine-induced H_2_S levels were significantly increased in the control group compared with baseline (C: 2.676 ± 0.146; Baseline: 1.459 ± 0.05; *p* < 0.001). This confirmed the assay. L-cysteine-induced endogenous H₂S levels were significantly lower in the IP group than in the control group (*p* = 0.04). However, there was no significant difference in H_2_S levels between the IP and IP-R groups (*p* > 0.99). Administration of NaHS before reperfusion significantly increased L-cysteine–induced H₂S levels compared with the IP-R group (IP-[NaHS]-R: 2.513 ± 0.036; IP-R: 2.32 ± 0.094; *p* = 0.0496) (Fig. 7b).

## Discussion

The present study demonstrated the role of H_2_S in experimentally induced IP in rats. After a 4-hour ischemic period, the penile tissues of the IP group exhibited vasocongestion, desquamation, edema, inflammatory cell infiltration, and increased collagen deposition. These findings were also observed in the IP-R group. L-cysteine-induced H₂S formation was diminished in parallel with increased HIF-1α levels in these groups. Intraperitoneal administration of the H₂S donor NaHS prior to reperfusion significantly reduced vasocongestion, collagen deposition, and desquamation. In contrast, edema and inflammatory cell infiltration were not markedly affected. NaHS treatment significantly increased H₂S levels and reduced HIF-1α expression compared to the IP-R group.

Ischemia in penile tissue leads to several structural changes that contribute to erectile dysfunction. These changes are primarily due to reduced blood flow and oxygen supply, resulting in tissue damage. Accumulation of reactive oxygen and nitrogen species prompts smooth muscle dysfunction and microvascular damage in the ischemic environment [22]. Ischemia is also associated with penile fibrosis, characterised by excessive collagen deposition, alterations in the extracellular matrix, inflammation, and edema in penile tissues [23]. The management of IP involves reperfusion, but this paradoxically increases tissue damage. Kolukcu et al. reported that reperfusion in penile tissue following ischemic events results in significant structural and biochemical changes, including inflammation, cellular alterations, and oxidative stress [18]. Consistent with these studies, we observed edema, inflammation, and increased collagen deposition in penile tissue after four hours of ischemia. All of this ischemia-induced damage persisted into the reperfusion period. In addition to these stromal and vascular alterations, we observed urethral epithelial desquamation following I/R. Although urethral epithelial desquamation is not a commonly reported or defining feature of IP, ischemia-related epithelial shedding has been reported in intestinal epithelium exposed to hypoxic or ischemic stress increases intestinal permability [24]. In the present study, this finding is therefore interpreted as a secondary epithelial response to I/R stress, rather than as a primary pathological feature of IP.

HIF-1α is the primary factor that enables cells and tissues to adapt to low oxygen levels by activating genes essential for their survival and function. However, HIF-1α can both cause and prevent tissue damage; its effects appear to depend largely on the tissue type, disease context, and severity of hypoxia. An in vivo study inducing renal tubular injury in rats via I/R reported that HIF-1α accumulates under hypoxic conditions during ischemia and stabilizes during the reperfusion phase. It has also been suggested that stabilisation of HIF-1α expression during reperfusion protects proximal tubules and contributes to their regeneration [25]. Nevertheless, HIF-1α has been reported to induce inflammation and oxidative stress under high-glucose and hypoxic conditions, thereby contributing to vascular injury in endothelial cells [26]. Increased HIF-1α expression in penile tissue has also been associated with the pathophysiology of priapism. An experimental study of priapism in a sickle cell anaemia mouse model revealed impaired in vivo erectile responses, increased HIF-1α gene expression, and decreased PDE5 gene expression in penile tissues, mediated by adenosine A₂B receptor activation [7]. Although the underlying mechanism was not investigated in the present study, we observed that HIF-1α expression in penile tissue increased in parallel with tissue injury during both phases of I/R in a rat model of ischemic priapism. Further research is required to elucidate the exact mechanisms involved. Taken together, these findings suggest that increased HIF-1α expression in IP may contribute to both functional and structural alterations in the corpus cavernosum.

Although several studies have examined the effects of H₂S in different tissues under ischemic conditions [10, 27, 28], to the best of our knowledge, no study has yet investigated its role in IP. The literature presents contradictory findings regarding H₂S levels under hypoxic conditions. For example, hypoxia has been reported to enhance H₂S production in carotid body glomus cells by upregulating cystathionine-γ-lyase (CSE) activity [27]. In contrast, studies on hypoxic Alzheimer’s transgenic mice demonstrated significantly reduced H₂S levels in the cerebral cortex, which were attributed to downregulation of cystathionine-β-synthase (CBS) [29]. Consistent with these findings, another study reported decreased H₂S levels in plasma and lung tissue in a hypoxia-induced pulmonary hypertension model [30]. Moreover, Tao et al. demonstrated that hypoxia significantly increased 3-mercaptopyruvate sulfurtransferase (3-MST) protein levels, decreased CSE protein levels, and had no effect on CBS protein levels, emphasising that these three H₂S-producing enzymes respond differently to hypoxic conditions [31]. Collectively, these findings suggest that the regulation of H₂S under hypoxia is highly tissue- and disease-specific, depending on the relative contributions and adaptive responses of CSE, CBS, and 3-MST. In our study, we found that H_2_S levels induced by L-cysteine in penile tissue decreased in the IP group under hypoxic conditions, and this reduction persisted throughout the reperfusion period. Another limitation of our study is that we were unable to determine changes in the levels of H₂S-synthesizing enzymes in ischemic priapism.

Exogenous administration of H₂S has been shown to protect tissues from I/R injury [9]. These studies reported that H₂S donors, applied at different concentrations, reduced IR-related damage by mitigating oxidative stress, inflammation, and apoptosis across multiple organs [9]. In our study, the intraperitoneal administration of the H₂S donor NaHS prior to reperfusion markedly attenuated vasocongestion, collagen deposition, and desquamation. However, it did not have a significant effect on edema or inflammatory cell infiltration. A possible explanation for this finding is that there was a tendency, although not statistically significant, toward reduced parameters of tissue damage (e.g., desquamation, vasocongestion, and collagen accumulation) during reperfusion, whereas inflammation and edema tended to increase. Exogenous administration of NaHS prior to reperfusion may have been insufficient to suppress these inflammatory alterations. It is also possible that achieving a more pronounced anti-inflammatory effect would require an alternative dosing strategy, such as repeated administration or a higher dose. This interpretation is supported by studies showing that the anti-inflammatory efficacy of H₂S donors varies with both dose and timing [32]. In a previous experimental study conducted in obese mouse models, H₂S was shown to exert cytoprotective effects in the corpus cavernosum by reducing oxidative stress–related tissue injury, and to preserve the structural integrity of erectile tissue [11]. This prior evidence is consistent with the tissue-protective effects observed with NaHS in the present study. Importantly, prior in vivo studies have demonstrated that intracavernosal administration of NaHS or Na₂S produces dose-dependent increases in intracavernosal pressure (ICP) and AUC (area under the curve), which reflects the total erectile response and prolongs erectile responses in anesthetized rats [33]. In addition, human corpus cavernosum studies have confirmed H₂S-mediated smooth muscle relaxation [34]. These findings highlight an important safety consideration in IP. Acute ischemic priapism is a urologic emergency in which rapid detumescence is the cornerstone of management, as emphasized in the AUA/SMSNA Guideline (2022) [35]. Therefore, although NaHS attenuated early reperfusion-associated tissue injury in our model, functional endpoints related to priapism resolution, such as ICP monitoring and time-to-detumescence analyses, were not evaluated and remain essential for future translational safety assessment.

We also showed that NaHS treatment decreased HIF-1α levels. Similar to our results, Kai et al. observed that 1 mM NaHS decreased HIF-1α protein accumulation and the expression of its downstream genes under hypoxic conditions in mammalian cells [36]. Another supporting study showed that NaHS treatment at relatively low concentrations (10–100 µM) reduced HIF-1α protein levels in HEK293T, Hep3B, and EA hy926 cells under hypoxic conditions [37]. Impaired HIF-1α signalling has been implicated in priapism-induced erectile dysfunction, suggesting a complex interplay among hypoxia, HIF-1α, and erectile physiology [7]. Since HIF-1α upregulation contributes to cellular processes such as inflammation and fibrosis, the reduced reperfusion injury observed in the IP-R group in our study may be associated with H₂S-mediated downregulation of HIF-1α.

Effective treatment of IP requires restoration of corporal venous outflow and arterial inflow to increase corporal partial pressure of oxygen [6]. First-line treatment involves diagnostic aspiration followed by either therapeutic aspiration, aspiration/irrigation, or injection of a sympathomimetic drug. Reperfusion of penile tissue with oxygenated blood is necessary for effective smooth muscle contraction in response to sympathomimetic drugs (phenylephrine, ephedrine, epinephrine, norepinephrine, metaraminol) [2]. A significant increase in oxygen levels during restoration of cavernous-body blood flow has been shown to exacerbate tissue damage in IP treatment [6]. In our study, we also demonstrated that tissue damage persisted in the reperfusion group. Therefore, we administered NaHS treatment before reperfusion in our study.

## Conclusion

The present study demonstrated a decrease in H₂S levels during the 4-hour ischemia and subsequent reperfusion period; this decrease paralleled tissue damage characterised by edema, inflammation, desquamation, vasocongestion, and increased collagen accumulation in the penile tissue, indicating the involvement of H₂S in the pathophysiology of IP. Administration of the H₂S donor, NaHS before reperfusion reduced both tissue damage and HIF-1α levels.

Our findings also demonstrated that HIF-1α expression increased in parallel with the severity of tissue injury during both ischemia and the early reperfusion phase. Although the precise mechanisms linking HIF-1α activation to corporal tissue injury remain to be fully elucidated, these findings suggest that HIF-1α may reflect hypoxia- and reperfusion-associated cellular stress rather than functional outcomes related to the resolution of priapism. In addition, our data indicate that NaHS may serve as a potential tissue-protective adjunct by attenuating reperfusion-associated injury following IP. However, given that H₂S donors have been reported to enhance erectile responses and increase ICP, future investigations should incorporate detumescence time and ICP measurements as essential safety endpoints to exclude the risk of prolonged priapism, as prompt detumescence constitutes the cornerstone of guideline-based management of acute ischemic priapism.

## Data Availability

All data supporting the findings of this study are available within the paper.
